# Basic Pan-Cancer Analysis of the Carcinogenic Effects of Cyclin-Dependent Kinase 4 (CDK4) in Human Surface Tumors

**DOI:** 10.1155/2021/8493572

**Published:** 2021-08-09

**Authors:** Jingping Wu, Tinghan Deng, Yuanen Huang, Hongbin Cheng

**Affiliations:** ^1^Department of Medical Cosmetology, Hospital of Chengdu University of Traditional Chinese Medicine, Chengdu 610075, China; ^2^Dermatology of Department, Hospital of Chengdu University of Traditional Chinese Medicine, Chengdu 610075, China

## Abstract

Although the evidence based on current human, animal, or molecular biology can explain some of the relationships between CDK4 and cancer, there is no pan-cancer analysis of the gene CDK4 in human skin tumors. Therefore, the potential carcinogenic effects of CDK4 in 33 tumors were initially explored in the datasets of the GEO (Gene Expression Omnibus) and the CGA (Cancer Genome Atlas). We found that CDK4 was highly expressed in most cancers and that CDK4 performance levels significantly correlated with the prognosis of cancer patients. These were found in our preliminary exploration. In addition, we used the dataset in tumors such as cutaneous melanoma or lung adenocarcinoma and found increased levels of phosphorylation of r24 l/C/h/s. In addition, fibroblast infiltration associated with CDK4 cancer was observed in head and neck, sarcoma, and melanoma skin. Using this pan-cancer study, our group has provided a comprehensive preliminary demonstration of the oncogenic effects of the CDK4 gene on different human skin tumors.

## 1. Introduction

As we all know, tumorigenesis and development is a very complicated and difficult to study process. Therefore, for cancer research, we conduct pan-cancer analysis based on the genes we are interested in and evaluate them, including the relevance of clinical prognosis, and potential various contents including molecular mechanism are very significant. There are many tumor genomic datasets in the CGA (Cancer Genome Atlas) project and the existing GEO dataset on which we can perform pan-cancer analysis [1–3]. In many tumors, apoptosis resistance is an important cause of drug insensitivity and chemotherapy resistance. With the deepening of the understanding of tumor pathogenesis, molecular targeted therapy has also been continuously developed, among which antiapoptotic pathways are an important therapeutic strategy. The B-cell leukemia-2 family is a protein family responsible for regulating cell apoptosis. It contains more than 20 proteins, which can be divided into antiapoptotic proteins and proapoptotic proteins according to their functions. Some studies have proposed biomarkers that may be used as cancer predictors, Wnt signal pathway, the carbohydrate metabolism signal pathway, and the PI3K-Akt signal pathway; in fact, there are some similarities with our research [4].

RNP2 is a protein complex that catalyzes DNA transcription in eukaryotes and can produce messenger RNA (mRNA), small nuclear RNA (snRNA), and precursors of microRNA. These processes are driven by general transcription factors, including TFIIA, TFIIB, TFIID, TFIIE, TFIIF, and TFIIH. Among these GFTs, TFIIH plays multiple functions in the transcription of various protein-coding genes and DNA nucleotide excision repair pathways. TFIIH is a protein complex containing 10 subunits, of which 7 subunits form the core complex. The CAK subcomplex is composed of CDK7, MALT, and cyclin H. Cyclin-dependent kinase is the serine/threonine kinase section of the CyclinD and the DC complex; its members are of the protein family. Since its discovery in 2013, life cycle proteins G and H and catalytic subunits, molecules that regulate kinase activity in cells, have been extensively studied [5]. The mammalian cell cycle is driven by cyclin and its related cyclin-dependent kinase (CDK). From the existing research on the cell cycle mechanism, it can be known that D-type cyclin and its related CDK (CDK4 and CDK6) are one of the key steps. They can promote the transition from G2 to M phase [6]. In the meantime, the cyclin CDK axis plays an irreplaceable role: controlling proliferation, senescence, migration, apoptosis, and angiogenesis [7]. In different types of tumor cells, the expression of CDKs is often unregulated, which makes them a potential target for cancer treatment. A series of drugs targeting different CDKs have been extracted or synthesized from natural plants. These inhibitors can be divided into two categories according to their mechanism of action: competitive inhibitors and covalent inhibitors. CDK7 inhibitors show significant antitumor activity in hematological tumors and a variety of solid tumors. Some of the most effective CDK7 inhibitors have IC50s of less than 200 nM in a large number of cells. In the past two years, some CDK7 inhibitors have entered phase I clinical trials.

This paper analyzes the pan-cancer research on CDK4 through the CGA medical results and GEO data validation set. The analysis includes gene mutation characteristics, menstrual cycle prognosis, genetic variation, protein phosphoric acid characteristics, immunotherapy, and related cancer cell lesions. Through analytical chemical analysis, the results of CDK4 in the clinical prognosis or pathogenesis of different cancers were verified.

## 2. Experimental Equipment and Method

### 2.1. Cancer Analysis

First, we used the TIMER3 website. There is a “Gene_DE” module on the website. Enter “CDK4” in this module. Then use the dataset of this website to analyze the differences in CDK4 gene performance in different tumors and adjacent normal tissues. In various tumors, there are very limited normal tissue tumors or some abnormal tumors (such as CGALAML (acute myeloid leukemia) and CGAGBM (glioblastoma multiform)), which cannot be analyzed by the TIMER3 website alone. In response to this situation, we used the GEPIA5 language [8]. The GEPIA5 tool depicts the box shape of most normal and abnormal tissue cells and differentially analyzes the performance of normal tissue cells in the GTEY dataset. After obtaining the above analysis, our group used the cancer analysis module of HEPIA3 to describe the form of performance, some tumors at four pathological stages in some CGA projects. It is violin plots of CDK4 gene levels. Converted performance data per million transcripts were applied to box-line plots or violin plots.

### 2.2. Prognostic Analysis

Our group used GEPIA5's “Survival Map” module in survival and prognosis analysis [9]. Through this module, we obtained the DFS (disease-free survival) and OS (overall survival) saliency data of the CDK4 gene in all tumors included in CGA. The performance threshold is divided into high-performance and low-performance queues, with cut-off high (50%) and low cut-off (50%) values. The hypothesis test in GEPIA5 uses the log-rank test. At the same time, the survival chart uses the cancer analysis module of GEPIA5 to obtain tumors with high and low CDK4 performance.

### 2.3. Genetic Alteration Analysis

We use the CBP (cBioPortal) tool [10–12]. The first step is to select “CGA Cancer Pan-Cancer Research” in the in-depth research section, and the second step is to enter “CDK4” to query the changes in gene characteristics CDK4. Using the terminal illness analysis tool, we can observe the change of radiofrequency and genetic mutation nature and copy the result change results of all CGA terminal illnesses. In the “Mutations” module, the mutation sites of CDK4 and all the sites displayed in the protein structure diagram and three-dimensional structure can be understood by us. There is also a “Comparison” module in the CBP (cBioPortal) to obtain complete works, no disease, no treatment effect, and no disease cycle data of cancer cases in the CGA project, especially when the level of the CDK4 gene changes. Kaplan–Meier plots with logarithmic M-value are also generated.

We used the cBioPortal tool (https://www.cBioPortal.org). The first step was to select CGA Pan-Cancer Atlas study in the depth research section and the second step was to enter CDK4 to query the characteristics of genetically altered CDK4. In the terminal illness type section module, we can observe the change of radiofrequency and genetic mutation nature and copy the result change results for all CGA terminal illnesses. In the mutation module, we can see at a glance the mutation sites of CDK4 and all the sites shown in the protein structure map and three-dimensional architecture. CBP (cBioPortal) also has a comparison module to obtain overall, disease-free survival data for CGA project cancer cases, especially if the CDK4 gene level has changed.

### 2.4. Immune Penetration Analysis

To analyze the immune infiltration, this paper utilizes the gene mutation analysis module of the TIMER3 tool to explore the tumors included in the CGA project. The relationship between immune invasion and CDK4 gene performance can be clearly identified in tumor patients. We then selected cancer- and CD10+ T-cell-associated fibroblast immune cells and used the QUANTISEQ, CIBERSORT-ABC, TIMER, CIBERSORT, WORD-TOOL, EPIC, and MCPCOUNTER algorithms to estimate immune invasion. Biased correlation values and M-value were adjusted by the purity of the grades and then relevant test data were obtained. For ease of viewing, we used visual scatter plots and heat maps to present the results.

### 2.5. CDK4 Gene Analysis

This paper uses the https://string-db.org website tool, enters individual protein names, such as DK3, DK4, DK5, etc., and selects the organism, such as Homo sapiens. In addition, the following parameters have been set: (1) the minimum interaction score, which is the score we need (“low confidence (0.150)”); (2) the meaning of the edge of the network (“evidence”); (3) the interaction to be displayed and the maximum number of participants, no more than 100 interactors in a cell; (4) active source of interaction (“experimental”). At last, we obtained CDK4-binding proteins, which are available and verified by experiments.

After finding the binding protein we need, we use the SGA (Similar gene analysis) module of GEPIA5 to search all of the tumors and normal tissues of these tumors included in the CGA project, and then we get the top 100 and CDK4. Targeted genes are closely related to genes. At the same time, the “correlation analysis” module in GEPIA5 helped us analyze the Pearson correlation between CDK4 and selected genes. We applied log2 TPM to the dot plot. Results of correlation coefficients and M-value are displayed. The module that provides heat map data for the selected genes is the Gene_Corr module of TIMER3. This module provides both M-value and biased correlation CBN values in the carcinogenic gene grade correlation test for purity adjustment.

In this paper, we use the interactive Venn diagram viewer, jvenn, for cross-tabulation analysis, by which we can help to compare CDK4-binding genes and interacting genes [13]. Again based on this paper, we use KEGG pathway analysis for both datasets, which is derived from the Encyclopedia of Genome and Gene. Briefly, there are three steps: (1) DAVIDS is the dataset for visualization and annotation, to which we saved the gene list. (2) Set the markers for the selected species. (3) Graph the data describing the functional annotation. Finally, two *R* packages, namely, “tidy” and “ggplot5,” were used to enrich the visualization methods. At the same time, we applied the *R* package of cluster Profiler for Gene Ontology analysis, GO analysis, and gene analysis. We calculated BP, cell composition, CC, biological process, and MF by cnetplot function, setting parameters as follows: circle = *F*, color edge = *T*, and node_label = *T*. The three parts of molecular functions were visualized as cnetplots; then H language software (h-3.6.5, 128 bits) was used for analysis (*P* < 0.01).

## 3. Experimental Result

### 3.1. Gene Performance Data Analysis

**Figure 1:**
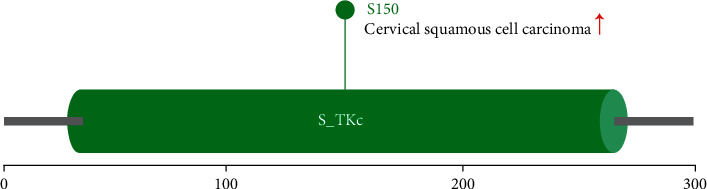
CDK4 protein phosphorylation analysis in tumors.

In the research process of this paper, we are committed to investigating the carcinogenic effect of human CDK4 (NM_000075.6 for protein or NP_000066.2 for mRNA). As shown in Figure 1, the structure of CDK4 protein in human species is usually composed of S_TKC (sm000220) domain. The evolutionary relationship of CDK4 between different species is shown through the Blast Tree View.

Our research group analyzed the CDK4 phosphoprotein (Y6, Y17, S52, S81, T149, S150, T172, S218, S285, and S294) performance levels between primary tissues of selected tumors and regular tissues through UALCAN. Based on the CPTAG dataset, the CDK4 protein diagram above shows the phosphoprotein sites with positive results. However, it is a pity that the increase in cellular squamous cell carcinoma was only found in S150, and the rest of the data were not searched in the UALCAN and CPTAG datasets.

**Figure 2:**
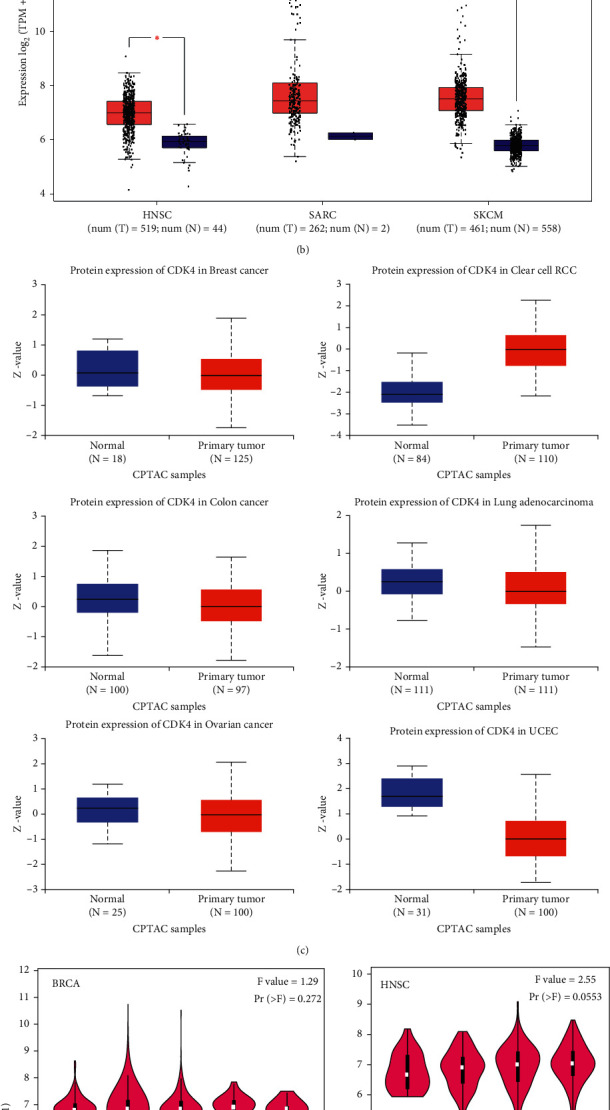
Results of CDK4 gene performance levels in different tumor pathological stages. (a) TCGA dataset. (b) TCGA + GTEx dataset. (c) CPTAC dataset. (d) TCGA dataset.

In this paper, we analyzed the performance pattern of CDK4 in nontumor tissues, and the experimental results are shown in Figure 2(a). We combined three datasets, FANTOM5 (Mammalian Genome Function Annotation (5), GTEY, and HPA (Human Protein Atlas). It was found that CDK4 performance was highest in the ovary, followed by the placenta and adrenal gland. It can also be found by Figure 2(a) that CDK4 performance in all detected tissues shows low RNA tissue specificity with all uniformly normalized performance values >1. In this paper, when analyzing the CDK4 performance of three different blood cells in the dataset Ph.B./Austria/Schimel, we also observed the gene specialist of the low red blood cell type. The performance status of the CDK4 gene in CGA of various cancer types was analyzed from TIMER3. As shown in Figure 2(a), renal clear cell carcinoma, THYM (thymoma) and UCE (uterine body endometrial), SKCM (cutaneous melanoma), STAD (gastric adenocarcinoma) (*P* < 0.01), SARC, READ (rectal adenocarcinoma) (*P* < 0.001), pulmonary adenopathy, prostate adenopathy, cell disease, mesotheliomas, paragangliomas, pancreatic adenopathy, squamous cell disease, glial disease, breast disease infiltrating disease, acute myeloid leukemia, renal papillary cell disease, suspicious renal cell disease, head and neck squamous cell disease, glioblastomas multiforme, esophagus disease, cholangiopathies, cervical squamous cell disease, cervical adenopathy, ovarian serous cystic adenopathy, bladder urolithiasis diffuse large B-cell lymphoma, colorectal adenopathy, and adrenocortical disease were all higher than the corresponding control tissues (*P* < 0.01).

The control group selected normal tissues in the GTEY dataset. From Figure 2(b), it can be seen that the differences in CDK4 performance between tumor tissues include SARC (Sarcoma), normal human surface tissue, SKCM (skin melanoma), and HNSC (head and neck). The results of the CPTAG dataset show that (Figure 2(c)) the total protein performance of CDK4 in normal tissues is lower than that of clear cell RCG, breast, ovarian, colon, LUAH and UCEG, and primary tissue (*P* < 0.005). The connectivity between the performance of CDK4 and the stage of cancer was observed using the pathological stage diagram module of HEPIA3 (Figure 2(d)), including BRCA, HNSC, OV, and SKCM (*P* < 0.1).

CDK4 gene performance status in specific cancer or different cancer subtype is analyzed by using the TIMER3 method (*P* < 0.1, *P* < 0.05, and *P* < 0.005). We analyzed three tumor types CGA items, SARC, THNSC, and SKCM, using existing methods for box-line plot data analysis, and normal tissue from the GTEY dataset was selected for the control group. When *P* < 0.05, based on the CMTAG dataset, we analyzed cell RC (renal cell), colon cancer, ovarian, breast, UCEG (endometrial), and UAD (lung adenocarcinoma). When *P* < 0.005, Log3 (TPM + 2) was chosen for logarithmic scale based on TCGB dataset to analyze the performance levels of SMD2 gene according to the I, II, III, and IV pathological stages of BRCA, HNSC, OV, and SKCM.

### 3.2. Survival Analysis Data

**Figure 3:**
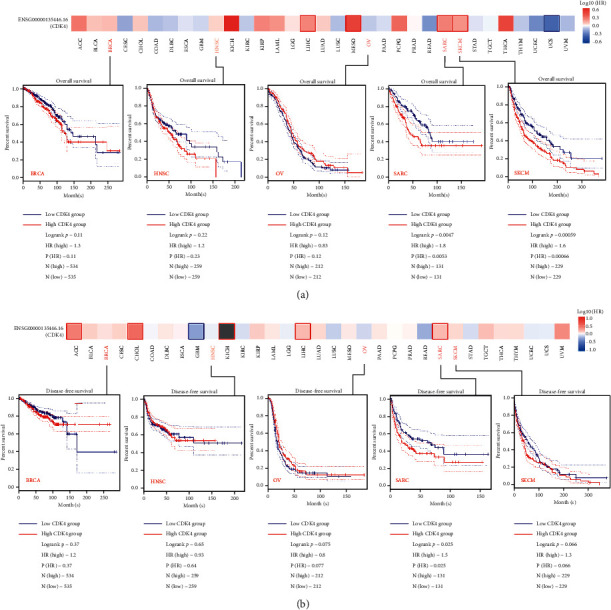
The description of the correlation between the CDK4 gene and cancer survival prognosis in the CGA project. (a) Overall survival. (b) Disease-free survival.

Cancer cases were divided into high- and low-performance experimental groups according to the level of performance of the CDK4 gene. The correlation between the patient with different tumors and the different performance of the CDK4 gene was investigated by analysis of GEO and CGA datasets. As shown in Figure 3, the high gene performance of CDK4 was correlated with poor prognosis of overall survival in OV cancer in the CGA project (*P* ≤ 0.12). The results of disease-free survival analysis demonstrated a strong correlation between high CDK4 gene performance and poor prognosis in CGA cases with OV (*P* < 0.075) and BRCA (*P* ≤ 0.37). In contrast, low CDK4 gene performance was associated with poor prognosis of disease-free survival in HNSC (*P* < 0.22), SARC (*p* ≤ 0.0047), SKCM (*p* < 0.00059), BRCA (*P* ≤ 0.11), SARC (*P* < 0.025), and KCM (*P* ≤ 0.066). For HNSC, the performance of the CDK4 gene may not have a significant effect on its prognosis. The performance of CDK4 is related to the prognostic performance of different tumor cases. This can be learned from the above data we analyzed.

The CDK4 gene performance was analyzed by the GEPIA5 tool. The BRCA, HNSC, OV, SARC, and SKCM in the CGA project were analyzed for disease-free survival (a) and total life cycle (b), and the survival map and Kaplan were obtained. The Meier curve was positive.

### 3.3. Genetic Data Change Analysis

**Figure 4:**
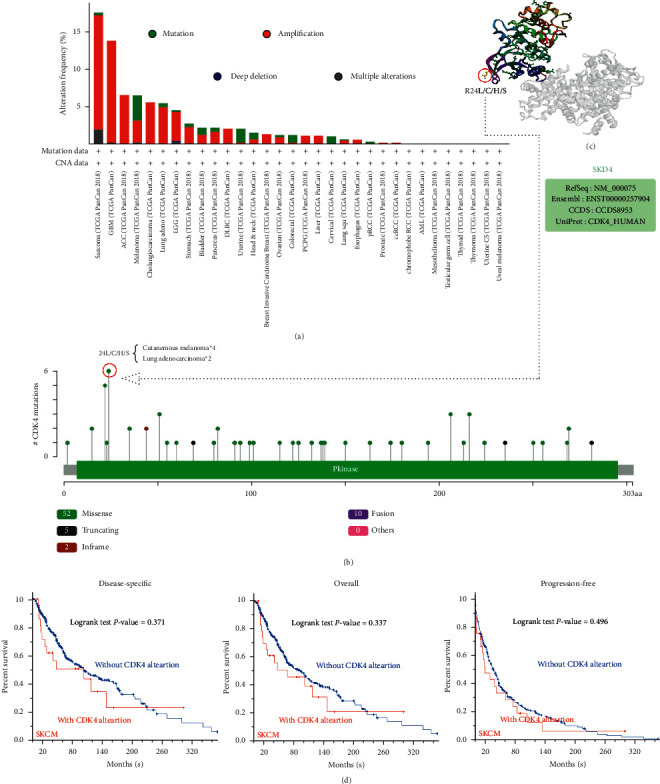
Results of CDK4 changes of CGA in different tumors.

In this part, different tumor data from the CGA cohort were selected to analyze the genetic pattern of CDK4.  Figure 4(a) shows the result; the highest change frequency (>15%) in CDK4 occurs in patients with sarcoma tumors whose main type is “amplification.” We found that the more prominent types in melanoma are the “amplification” type and the “mutation” type, which account for about 3.5% of the occurrence frequency. And we found an interesting phenomenon: most of the tumors listed in the picture are formed under the influence of CDK4, with the “amplification” type as the main type. The type, location, and number of cases in the genetic changes of the CDK4 gene are further shown in Figure 4(b). Through our data analysis, we identified missense mutations in CDK4 as the main type of genetic change. The R24 L/C/H/S changes of the Tudor domain have been detected in 4 cases of skin melanoma and 2 cases of lung adenocarcinoma (Figure 4(b)), and CDK4 gene is translated from R (arginine) to L (leucine)/C (cysteine)/H (histidine) at the 24th position of CDK4 protein/serine, and CDK4 protein is truncated. The R24 locus in the three-dimensional architecture of CDK4 protein is shown in Figure 4(c). Throughout the change pattern analysis, the potential relationship between CDK4 gene changes and prognosis of patients with different types of cancer was analyzed. The data in Figure 4(d) show that SKCM cases with altered CDK4 have overall (*P* < 0.337) and disease-specific (*P* < 0.371) survival rates and progression-free (*P* < 0.496) survival rates compared with cases without CDK4 changes. It showed a better prognosis.

We used the tool to analyze the CDK4 change pattern in CGA tumors. The change frequencies of mutation types (a) and mutation sites (b) are shown in Figure 4. The variety site with the highest change frequency (R24 L/C/H/S) is shown in the three-dimensional architecture of CDK4. Correlations between disease specificity and mutation status, overall SKCM, and free survival were also investigated using the tools in this paper.

### 3.4. Protein Phosphorylation Analysis Data

In this paper, we compare the phosphorylation of CDK4 between primary tumor tissue and normal tissue. We did not include relevant data in the dataset. Based on the CPTAG dataset, we analyzed the performance levels of CDK4 phosphoproteins (Y6, Y17, S52, S81, T149, S150, T172, S218, S285, and S294) between primary and normal tissue of the selected tumors by UALCAN. Positive results for phosphoprotein sites are shown by the CDK4 protein schematic. However, cellular squamous cell carcinoma increased intelligence was found in S150, and the others were not supported by the CPTAG and UALCAN datasets. The results of this experiment provide some ideas and approaches for further exploration of S150 phosphorylation in tumorigenesis.

### 3.5. Analysis of Immune Infiltration Data Results

**Figure 5:**
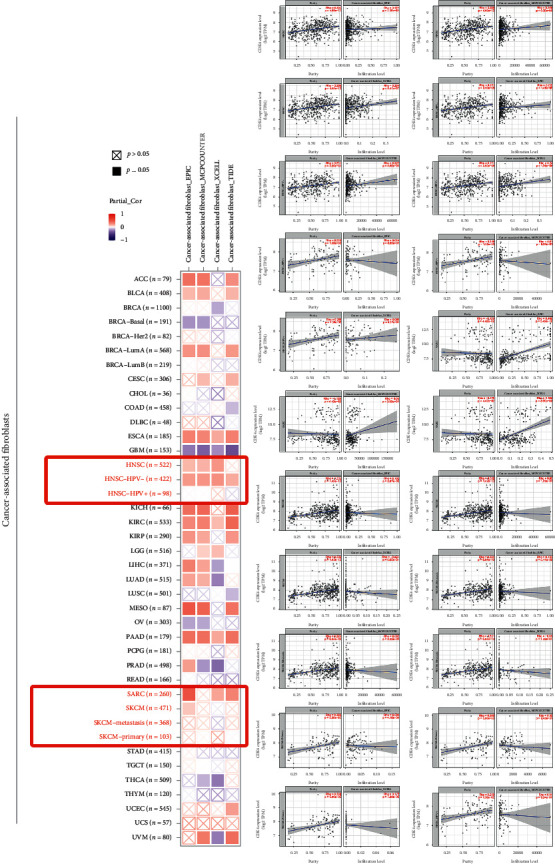
Further analysis of the correlation between the performance of CDK4 and the immune infiltration of cancer-related fibroblasts.

Tumor-infiltrating immune cells are closely associated with tumorigenesis, development, and metastasis [14, 15]. According to published literature, regulation of the functions of various tumor-infiltrating immune cells is mainly performed by tumor fibroblast in the tumor environment stroma [16, 17]. Based on existing studies, the correlation between CDK4 gene performance and different levels of immune cell infiltration in CGA of different cancer types was investigated using EXCELL, TIMER, QUANTISEQ, MCPCOUNTER, EPIC, and CIBERSORT-ABS algorithms. Through correlation studies, a statistically negative correlation between CD10+ T-cell immune infiltration and CDK4 performance in SARC, HNSC, and SKCM tumors was experimentally demonstrated (Figure S3).  Figure 5 shows the scatter data obtained by the algorithm for the above tumors. Different algorithms were used to explore all types of cancers in CGA to investigate the correlation between CDK4 gene performance levels and cancer cell infiltration levels.

### 3.6. CDK4 Enrichment Data Analysis

**Figure 6:**
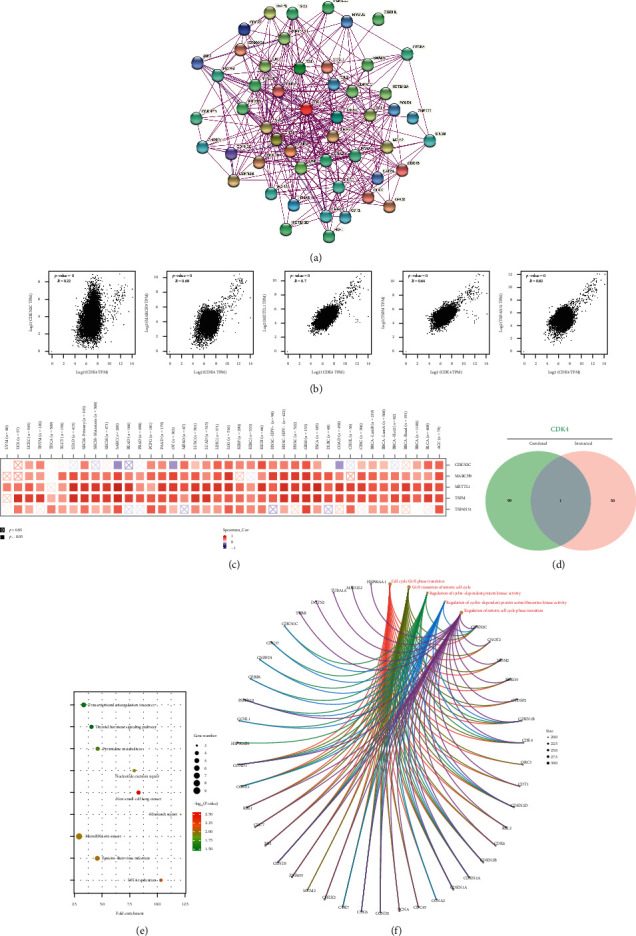
Gene enrichment analysis, CDK4.

In this paper, we analyze the molecular of CDK4 gene by studying the molecular, and we tried to screen the genes related to the performance of CDK4-binding protein and CDK4 and conducted a series of pathway enrichment analyses. Fifty CDK4-binding proteins were obtained, based on the STRING tool, which was supported by evidence. Interaction relationships of CDK4 cellular proteins are shown in Figure 6(a). Combining all tumor performance data from CGA, the high 200 genes associated with CDK4 performance were obtained using the GEPIA5 tool (Figure 6(b)). As seen in Figure 6, the cell cycle protein-dependent kinase inhibitor C2 was positively correlated with CDK4 performance levels (*R* = 0.22), METTL1 (Methyltransferase-Like 1) (*R* = 0.7), TSFM (Ts Translation Elongation Factor, Mitochondrial) (*R* = 0.64), and TSPAN31 (Tetraspanin 31) (*R* ≤ 0.81) genes (*P* < 0.002). Among the cancer types, a positive correlation between CDK4 and the genes mentioned above can be seen (Figure 6(c)). By cross-tabulating the data from the above two groups (Figure 6(d)), the experimental results showed that the two groups belonged to a common feature.

The important steps of KEGG and GO enrichment analysis require us to combine the two datasets to do. The KEGG data in Figure 6(e) show that the impact of CDK4 on tumor pathogenesis may be involved in two parts. By deeper analysis of the enrichment dataset, the experimental results demonstrated that most of the genes are closely related to cell biology (Figure 6(f)).

## 4. Discussion

Each stage of the cell cycle has a specific CDK/cyclin complex responsible for controlling the drive, and the activity of CDKs is induced by mitotic signals. When CDKs bind to their respective cyclins and are phosphorylated in the T-loop region, CDKs will be fully activated, and when DNA damage occurs, cell cycle checkpoints are activated, inhibiting the activity of CDKs. In cells, once various cell cycle-related proteins are abnormally activated, the cells will lose control and proliferate wantonly. This is the prerequisite for tumorigenesis and one of the characteristics of malignant tumors. Therefore, regulating or blocking the cell cycle is one of the important ways to treat tumors, and cell cycle-related regulatory factors are also considered to be valuable therapeutic targets.

The latest research shows that many CDK4 genes are closely related to clinical diseases, especially tumors [18–21]. How CDK4 plays a relevant role in different tumorigenesis through other molecular mechanisms, although many scholars have showed various new ideas, the exact mechanism has not been studied clearly. Through literature search, we have not found any research from the perspective of basic analysis of all tumors, especially from the perspective of human skin tumors, to retrieve the literature on CDK4 pan-cancer analysis. Based on this, this paper presents a comprehensive data analysis of the CDK4 gene in 35 different tumors based on data from different datasets such as CPTAG and CGA, while investigating the molecular characteristics of genetic changes and gene performance.

CDK4 has high gene performance in most tumors. From the above data, it is not difficult to see that striving for the downward adjustment of CDK4 in this direction is what we need to focus on. Tumorigenesis is a complex process involving many factors and links, which has not been fully studied at present, but according to the current data, the cell cycle plays an important regulatory role. For example, this paper uses another WEB server to perform gene regression physiological analyses on data from the SKCM, CGA-HNSC, and SARC cohorts. Through the analysis, we found that the high performance of CDK4 was a risk factor for both SARC and SKCM tumors, while no statistical significance was found for HNSC (SARC: Cox coefficient = 0.213, *P*=3.30*e* − 02; SKCN: Cox coefficient = 0.236, *P*=3.00*e* − 04; HNSC: Cox coefficient = 0.041, *P*=5.90*e* − 01). The key driver of cell cycle gene regulation is CDK4, which has an important role in the development of most malignancies [22]. Using the CGA project, in the skin field we are concerned about, three kinds of human body surface tumors, HNSC, SARC, and SKCM, are highly expressed in comparison with normal tissue progression experiments in the GTEY dataset. Therefore, in the next step, we can study the specific mechanism or pathway of CDK4 in the occurrence and development of related skin tumors to see if there is a new breakthrough or discovery.

The relationship between CDK4 gene performance and tumor survival and prognosis was also investigated in this paper. We analyzed the full physiological time and disease-free physiological time of CDK4 gene performance with a variety of tumors, giving positive physiological maps and gene change curves. It was found that HNSC, SARC, and SKCM showed high performance of CDK4 and decreased overall survival time in all concerned skin tumors, while in disease-free survival, there was no significant difference between high performance and low performance in HNSC, but the overall prognosis of low performance was better than that of high performance, while the disease-free survival time of high performance of SARC and SKCM was less than that of low performance. At this point, it can be suggested that the downregulation of CDK4 may be a good way to improve the prognosis of patients. As far as the current research is concerned, CDK inhibitors have played a great role in clinic, especially in breast cancer, which is the most studied at present [23, 24]. Some scholars have proposed a model-cloud-based intelligent BCP-T1F-SVM, which is specially used to define the stage and type of cancer patients suffer from [25]. If the relevant regulatory nodes for skin tumors can be found, the scope of application will be further expanded for the benefit of patients and clinical practice.

Among the mutational features of CDK4 in multiple tumors, the essential connection between overall SKCM and gene mutation, disease specificity, and the disease or progression physiology is of particular interest to us. Current studies have described numerous aberrations in gene cycle regulatory proteins in melanoma. The most important is the suppressor mutation of the CDK4 inhibitor-p17INK6a, which has been analyzed in most melanoma gene lines and primary melanoma samples. The germline mutation in CDK4 has been analyzed in hereditary melanoma families [21]. The CDK4 inhibitor PD0332992 has been analyzed by the FDB for the treatment of cancer and is in early clinical trials for a variety of solid tumors, including melanoma. Melanoma genes were found to be sensitive to CDK4 inhibitors in vivo. This sensitivity may depend on the performance levels of CDK4 and CDKN5A [26, 27]. For all tumors in our study group, especially those in the dataset, we analyzed information on CDK4 component and CDK4 performance genes and performed multiple enrichment analyzes and effects of metabolic pathways, RNA metabolism, and other processes. The paper used multiple methods to observe the statistical negative correlation between CDK4 performance and CD10 levels.

In addition to CDK7, THZ1 also has an inhibitory effect on CDK12/CDK13 at higher concentrations. This may be related to the similar structure of CDK12/CDK13 and CDK7, which also contain cysteine in the four residues of C312. By silencing CDK7 expression with siRNA, activity and proliferation of bile duct cancer cells were decreased, but this does not mean that THZ1 only exerts antitumor effects through CDK7 in all cholangiocarcinoma cells, especially in cholangiocarcinoma cells with higher IC50. It cannot be ruled out that THZ1 can also play an antibiliary tract cancer effect by inhibiting CDK12 and CDK13. In the field of skin oncology, we have studied less. According to other studies, there has been the first dataset of skin tumors of BCC and SK races in China [28]. In the following research, we will continue to make progress and update our analytical conclusions.

## 5. Conclusions

To sum up, the current studies on CDK4 and even CDK6 are mostly focused on breast cancer; for example, some studies have clarified the application of some CAD methods in the detection and diagnosis of breast cancer [29], and the research is relatively mature, so can there be a deeper breakthrough in the direction of body surface tumors? Further exploration is needed. The first CDK4 pan-cancer results suggest a close relationship between CDK4 performance and protein phosphorylation, clinical prognosis, tumor mutational load, immune cell infiltration, and gene mutation. Analysis from the perspectives of cell regulation, clinical case analysis, and clinical medication will help to understand the role of CDK4 in development.

Although THZ2 shows better pharmacokinetic stability, it is less tolerated in in vivo experiments, which is the reason why THZ1 was chosen for research. In order to make THZ1 have greater application prospects, it will be important to improve the molecular structure of THZ1, synthesize new THZ1 derivatives, improve its pharmacokinetic stability, and solve the limitations of the excessively high administration frequency of THZ1.

## Data Availability

The datasets generated and/or analyzed during the current study are available in the CGA repository, https://www.tcga.org/, GEO repository, https://earthobservations.org/index.php/, TIMER3 repository, http://timer.cistrome.org/, GEPIA5 repository, http://GEPIA5.cancer-pku.cn/#analysis/, CBP (cBioPortal) repository, https://www.CBP (cBioPortal).org/, STRING repository, https://string-db.org/, HPA repository, http://www.proteinatlas.org/, GTEY repository, https://www.genome.gov/Funded-Programs-Projects/Genotype-Tissue-Expression-Project/, FANTOM5 repository, https://fantom.gsc.riken.jp/5/, CPTAG repository, https://proteomics.cancer.gov/programs/cptac/, and UALCAN repository, http://ualcan.path.uab.edu/index.html/.

## Supplementary Materials

Supplementary Materials Supplement picture 1. The Blast Tree View of CDK4. The evolutionary relationship between CDK4 protein in different species is shown by Blast Tree View. Supplement picture 2. Analysis of the expression of CDK4 in human tissues and blood cells. (a) Based on the combination of HPA, GTEx, and FANTOM5, CDK4 is the highest expression in the ovary, followed by the placenta and adrenal glands, which we found. At the same time, CDK4 can be expressed in all detected tissues (all consistent normalized expression values >1) and show low RNA tissue specificity. (b) When analyzing CDK4 expression in different blood cells in the HPA/Monaco/Schmiedel dataset, low RNA blood cell type specificity will also appear. Supplement picture 3. Analysis of immune infiltration and CDK4 expression of CD8 + T cells in tumors. We observed a statistically negative correlation between CD8+ T-cell immune infiltration and CDK4 expression in SARC, HNSC, and SKCM tumors, which was verified by most algorithms.
