# Supplementary material for: Basic Pan-Cancer Analysis of the Carcinogenic Effects of Cyclin-Dependent Kinase 4 (CDK4) in Human Surface Tumors
Source: J Healthc Eng. 2021 Aug 9;2021:8493572. doi: 10.1155/2021/8493572 (PMC8371625; doi:10.1155/2021/8493572)
Supplement: Supplementary Materials — Supplement picture 1. The Blast Tree View of CDK4. The evolutionary relationship between CDK4 protein in different species is shown by Blast Tree View. Supplement picture 2. Analysis of the expression of CDK4 in human tissues and blood cells. (a) Based on the combination of HPA, GTEx, and FANTOM5, CDK4 is the highest expression in the ovary, followed by the placenta and adrenal glands, which we found. At the same time, CDK4 can be expressed in all detected tissues (all consistent normalized expression values >1) and show low RNA tissue specificity. (b) When analyzing CDK4 expression in different blood cells in the HPA/Monaco/Schmiedel dataset, low RNA blood cell type specificity will also appear. Supplement picture 3. Analysis of immune infiltration and CDK4 expression of CD8 + T cells in tumors. We observed a statistically negative correlation between CD8+ T-cell immune infiltration and CDK4 expression in SARC, HNSC, and SKCM tumors, which was verified by most algorithms. [file 8493572.f1.zip › 8493572.f1/Supplement picture 2a.pdf]

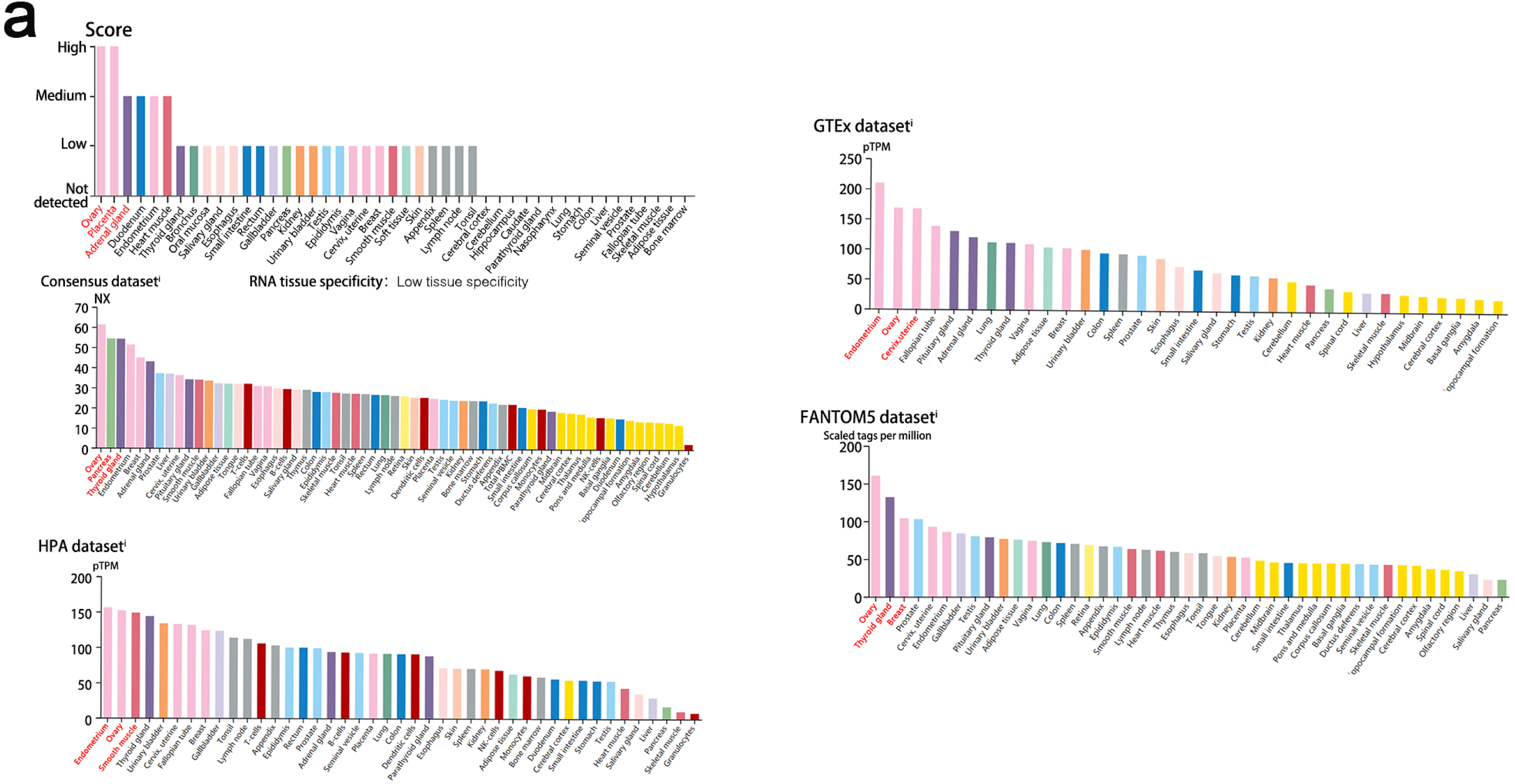
a
Score
High
Medium
Low
Not detected
Ovary
Placenta
Adrenal gland
Duodenum
Endometrium
Heart muscle
Thyroid gland
Bronchus
Oral mucosa
Salivary gland
Esophagus
Small intestine
Rectum
Gallbladder
Pancreas
Kidney
Urinary bladder
Testis
Epididymis
Vagina
Cervix, uterine
Breast
Smooth muscle
Soft tissue
Skin
Appendix
Spleen
Lymph node
Tonsil
Cerebral cortex
Cerebellum
Hippocampus
Caudate
Parathyroid gland
Nasopharynx
Lung
Stomach
Colon
Liver
Seminal vesicle
Prostate
Fallopian tube
Skeletal muscle
Adipose tissue
Bone marrow
Consensus datasetⁱ
RNA tissue specificity： Low tissue specificity
NX
70
60
50
40
30
20
10
0
Ovary
Pancreas
Thyroid gland
Endometrium
Breast
Adrenal gland
Prostate
Liver
Cervix, uterine
Pituitary gland
Smooth muscle
Urinary bladder
Gallbladder
Adipose tissue
Tongue
T-cells
Fallopian tube
Vagina
Esophagus
B-cells
Salivary gland
Thymus
Colon
Epididymis
Skeletal muscle
Tonsil
Heart muscle
Spleen
Rectum
Lung
Lymph node
Retina
Skin
Dendritic cells
Placenta
Testis
Seminal vesicle
Kidney
Bone marrow
Stomach
Ductus deferens
Appendix
Total PBMC
Small intestine
Corpus callosum
Monocytes
Parathyroid gland
Midbrain
Cerebral cortex
Thalamus
Pons and medulla
NK-cells
Basal ganglia
Duodenum
Hippocampal formation
Amygdala
Olfactory region
Spinal cord
Cerebellum
Hypothalamus
Granulocytes
HPA datasetⁱ
pTPM
200
150
100
50
0
Endometrium
Ovary
Smooth muscle
Thyroid gland
Urinary bladder
Cervix, uterine
Fallopian tube
Breast
Gallbladder
Tonsil
Lymph node
T-cells
Appendix
Epididymis
Rectum
Prostate
Adrenal gland
B-cells
Seminal vesicle
Placenta
Lung
Colon
Dendritic cells
Parathyroid gland
Esophagus
Skin
Spleen
Kidney
NK-cells
Adipose tissue
Monocytes
Bone marrow
Duodenum
Cerebral cortex
Small intestine
Stomach
Testis
Heart muscle
Salivary gland
Liver
Pancreas
Skeletal muscle
Granulocytes
GTEx datasetⁱ
pTPM
250
200
150
100
50
0
Endometrium
Ovary
Cervix,uterine
Fallopian tube
Pituitary gland
Adrenal gland
Lung
Thyroid gland
Vagina
Adipose tissue
Breast
Urinary bladder
Colon
Spleen
Prostate
Skin
Esophagus
Small intestine
Salivary gland
Stomach
Testis
Kidney
Cerebellum
Heart muscle
Pancreas
Spinal cord
Liver
Skeletal muscle
Hypothalamus
Midbrain
Cerebral cortex
Basal ganglia
Amygdala
Hippocampal formation
FANTOM5 datasetⁱ
Scaled tags per million
200
150
100
50
0
Ovary
Thyroid gland
Breast
Prostate
Cervix, uterine
Endometrium
Gallbladder
Testis
Pituitary gland
Urinary bladder
Adipose tissue
Vagina
Lung
Colon
Spleen
Retina
Appendix
Epididymis
Smooth muscle
Lymph node
Heart muscle
Thymus
Esophagus
Tonsil
Tongue
Kidney
Placenta
Cerebellum
Midbrain
Small intestine
Thalamus
Pons and medulla
Corpus callosum
Basal ganglia
Ductus deferens
Seminal vesicle
Skeletal muscle
Hippocampal formation
Cerebral cortex
Amygdala
Spinal cord
Olfactory region
Liver
Salivary gland
Pancreas
