# Supplementary figures and images for: Basic Pan-Cancer Analysis of the Carcinogenic Effects of Cyclin-Dependent Kinase 4 (CDK4) in Human Surface Tumors
Source: J Healthc Eng. 2021 Aug 9;2021:8493572. doi: 10.1155/2021/8493572 (PMC8371625; doi:10.1155/2021/8493572)

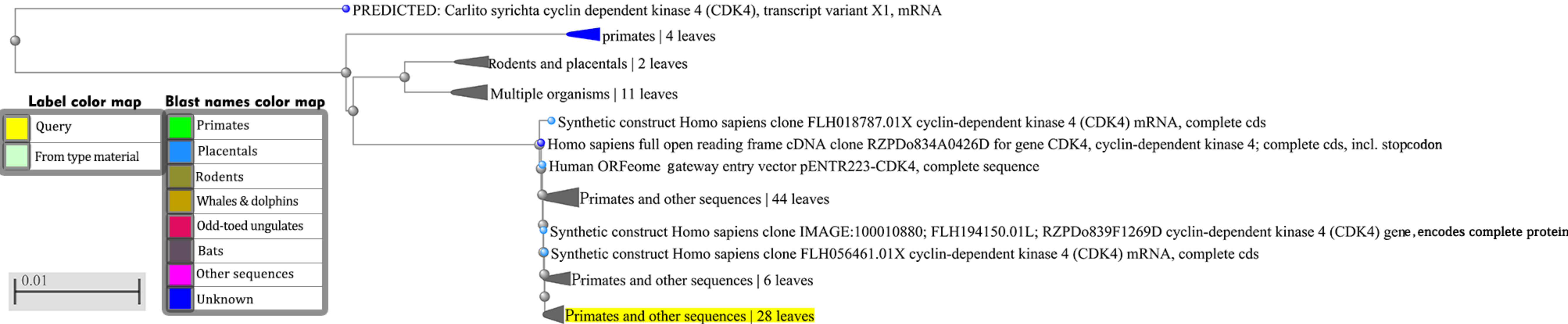

Supplement: Supplementary Materials — Supplement picture 1. The Blast Tree View of CDK4. The evolutionary relationship between CDK4 protein in different species is shown by Blast Tree View. Supplement picture 2. Analysis of the expression of CDK4 in human tissues and blood cells. (a) Based on the combination of HPA, GTEx, and FANTOM5, CDK4 is the highest expression in the ovary, followed by the placenta and adrenal glands, which we found. At the same time, CDK4 can be expressed in all detected tissues (all consistent normalized expression values >1) and show low RNA tissue specificity. (b) When analyzing CDK4 expression in different blood cells in the HPA/Monaco/Schmiedel dataset, low RNA blood cell type specificity will also appear. Supplement picture 3. Analysis of immune infiltration and CDK4 expression of CD8 + T cells in tumors. We observed a statistically negative correlation between CD8+ T-cell immune infiltration and CDK4 expression in SARC, HNSC, and SKCM tumors, which was verified by most algorithms. [file 8493572.f1.zip › 8493572.f1/Supplement picture 1.pdf]

b

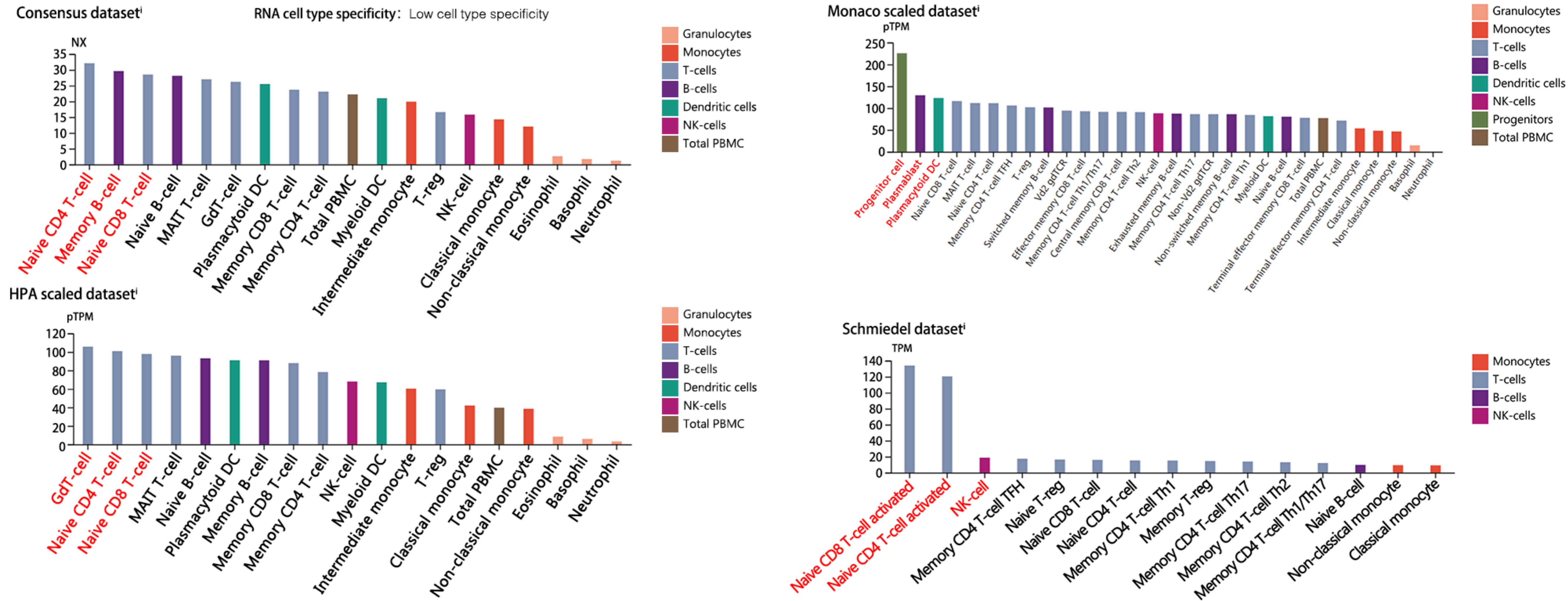

Supplement: Supplementary Materials — Supplement picture 1. The Blast Tree View of CDK4. The evolutionary relationship between CDK4 protein in different species is shown by Blast Tree View. Supplement picture 2. Analysis of the expression of CDK4 in human tissues and blood cells. (a) Based on the combination of HPA, GTEx, and FANTOM5, CDK4 is the highest expression in the ovary, followed by the placenta and adrenal glands, which we found. At the same time, CDK4 can be expressed in all detected tissues (all consistent normalized expression values >1) and show low RNA tissue specificity. (b) When analyzing CDK4 expression in different blood cells in the HPA/Monaco/Schmiedel dataset, low RNA blood cell type specificity will also appear. Supplement picture 3. Analysis of immune infiltration and CDK4 expression of CD8 + T cells in tumors. We observed a statistically negative correlation between CD8+ T-cell immune infiltration and CDK4 expression in SARC, HNSC, and SKCM tumors, which was verified by most algorithms. [file 8493572.f1.zip › 8493572.f1/Supplement picture 2b.pdf]

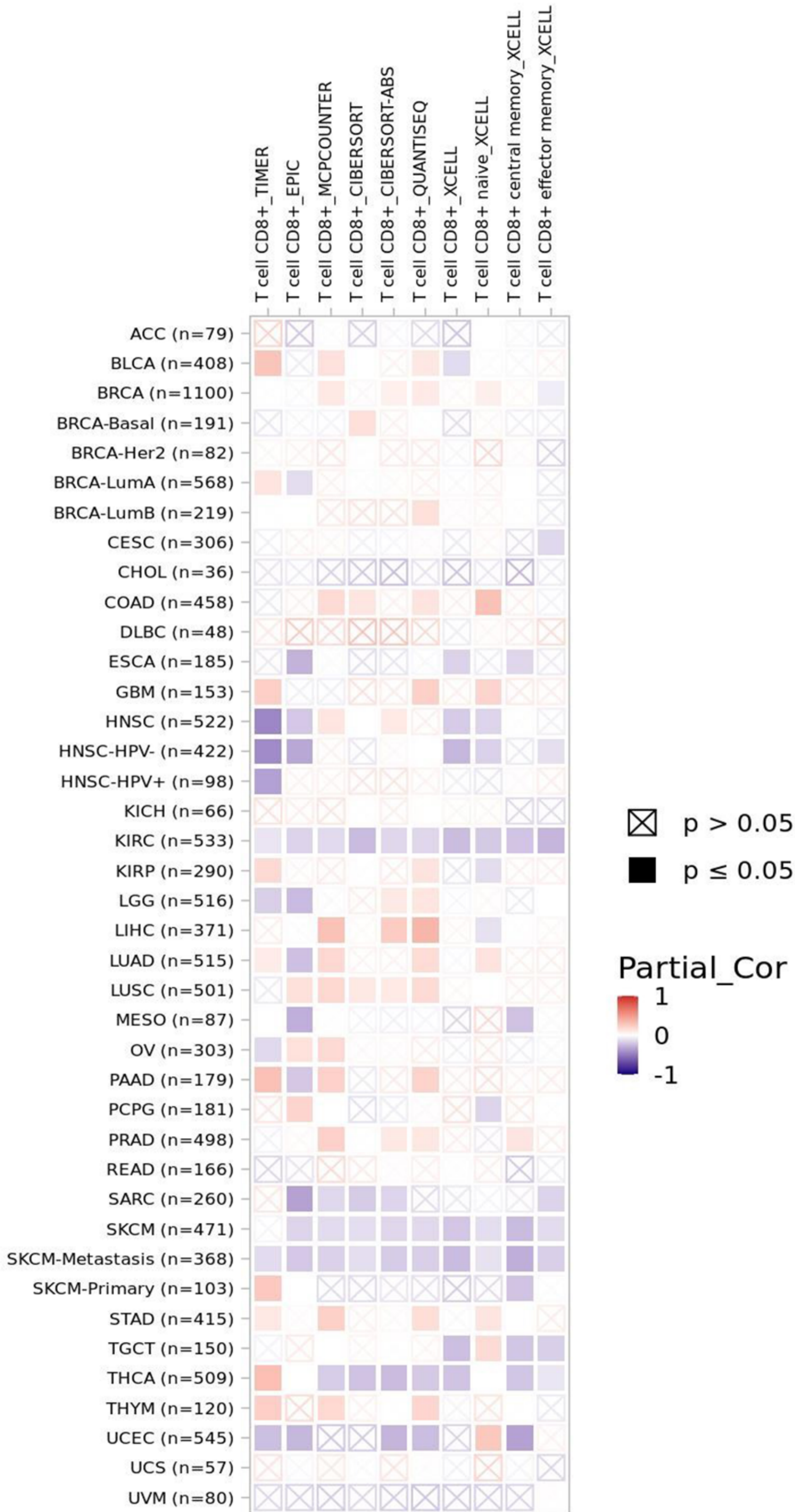

Supplement: Supplementary Materials — Supplement picture 1. The Blast Tree View of CDK4. The evolutionary relationship between CDK4 protein in different species is shown by Blast Tree View. Supplement picture 2. Analysis of the expression of CDK4 in human tissues and blood cells. (a) Based on the combination of HPA, GTEx, and FANTOM5, CDK4 is the highest expression in the ovary, followed by the placenta and adrenal glands, which we found. At the same time, CDK4 can be expressed in all detected tissues (all consistent normalized expression values >1) and show low RNA tissue specificity. (b) When analyzing CDK4 expression in different blood cells in the HPA/Monaco/Schmiedel dataset, low RNA blood cell type specificity will also appear. Supplement picture 3. Analysis of immune infiltration and CDK4 expression of CD8 + T cells in tumors. We observed a statistically negative correlation between CD8+ T-cell immune infiltration and CDK4 expression in SARC, HNSC, and SKCM tumors, which was verified by most algorithms. [file 8493572.f1.zip › 8493572.f1/Supplement picture 3.pdf]
